# Supplementary material for: Mixed methods protocol to examine the acceptability and clinical characteristics of a remote monitoring programme for delivery of COVID-19 care, among healthcare staff and patients
Source: BMJ Open. 2021 Sep 29;11(9):e051408. doi: 10.1136/bmjopen-2021-051408 (PMC8482534; doi:10.1136/bmjopen-2021-051408)
Supplement: Author's manuscript [file bmjopen-2021-051408.draft_revisions.pdf]

BMJ Open

BMJ Open is committed to open peer review. As part of this commitment we make the peer review history of every article we publish publicly available.

When an article is published we post the peer reviewers' comments and the authors' responses online. We also post the versions of the paper that were used during peer review. These are the versions that the peer review comments apply to.

The versions of the paper that follow are the versions that were submitted during the peer review process. They are not the versions of record or the final published versions. They should not be cited or distributed as the published version of this manuscript.

BMJ Open is an open access journal and the full, final, typeset and author-corrected version of record of the manuscript is available on our site with no access controls, subscription charges or pay-per-view fees (http://bmjopen.bmj.com).

If you have any questions on BMJ Open's open peer review process please email info.bmjopen@bmj.com

BMJ Open

Patient and staff acceptability and clinical characteristics of a remote monitoring programme for delivery of COVID-19 care.

Journal:	*BMJ Open*
Manuscript ID	bmjopen-2021-051408
Article Type:	Protocol
Date Submitted by the Author:	18-Mar-2021

Complete List of Authors:	Fox, Robert; University College Dublin, School of Nursing, Midwifery & Health Systems<br>Symmons, Sophie Mulcahy; University College Dublin, School of Nursing, Midwifery & Health Systems<br>De Brún, Aoife; University College Dublin, School of Nursing, Midwifery & Health Systems<br>Joyce, David; University College Dublin, School of Nursing, Midwifery & Health Systems<br>Muldoon, Eavan G; Mater Misericordiae University Hospital, Dept of Infectious Diseases; University College Dublin, School of Medicine<br>McGinty, Tara; Mater Misericordiae University Hospital, Infectious Diseases; University College Dublin, School of Medicine<br>O'Reilly, Katherine M.A.; University College Dublin, School of Medicine; Mater Misericordiae University Hospital, Department of Respiratory Medicine<br>O’Connor, Eileen; Mater Misericordiae University Hospital, Dept of Infectious Diseases<br>McAuliffe, Eilish; University College Dublin, School of Nursing, Midwifery and Health Systems

Keywords:	COVID-19, Telemedicine < BIOTECHNOLOGY & BIOINFORMATICS, PUBLIC HEALTH

SCHOLARONE™
Manuscripts

I, the Submitting Author has the right to grant and does grant on behalf of all authors of the Work (as defined in the below author licence), an exclusive licence and/or a non-exclusive licence for contributions from authors who are: i) UK Crown employees; ii) where BMJ has agreed a CC-BY licence shall apply, and/or iii) in accordance with the terms applicable for US Federal Government officers or employees acting as part of their official duties; on a worldwide, perpetual, irrevocable, royalty-free basis to BMJ Publishing Group Ltd ("BMJ") its licensees and where the relevant Journal is co-owned by BMJ to the co-owners of the Journal, to publish the Work in this journal and any other BMJ products and to exploit all rights, as set out in our licence.

The Submitting Author accepts and understands that any supply made under these terms is made by BMJ to the Submitting Author unless you are acting as an employee on behalf of your employer or a postgraduate student of an affiliated institution which is paying any applicable article publishing charge ("APC") for Open Access articles. Where the Submitting Author wishes to make the Work available on an Open Access basis (and intends to pay the relevant APC), the terms of reuse of such Open Access shall be governed by a Creative Commons licence – details of these licences and which Creative Commons licence will apply to this Work are set out in our licence referred to above.

Other than as permitted in any relevant BMJ Author's Self Archiving Policies, I confirm this Work has not been accepted for publication elsewhere, is not being considered for publication elsewhere and does not duplicate material already published. I confirm all authors consent to publication of this Work and authorise the granting of this licence.

For peer review only

Patient and staff acceptability and clinical characteristics of a remote monitoring programme for delivery of COVID-19 care.

Robert Fox[a], Sophie Mulcahy Symmons[a], Aoife DeBrun[a], David Joyce[a], Eavan G Muldoon[b,c], Tara McGinty[b,c], Katherine M.A.O'Reilly [c,d] Eileen O'Connor[b], Eilish McAuliffe[a]

[a]Centre for Interdisciplinary Research, Education and Innovation in Health Systems (IRIS), School of Nursing, Midwifery & Health Systems, University College Dublin

[b]Dept of Infectious Diseases, Mater Misericordiae University Hospital, Dublin

[c]School of Medicine, University College Dublin

d Department of Respiratory Medicine, Mater Misericordiae University Hospital

Author Note

Corresponding author: Robert Fox, Centre for Interdisciplinary Research, Education and Innovation in Health Systems (IRIS), School of Nursing, Midwifery & Health Systems, University College Dublin, Dublin, Ireland.
Email: Robert.Fox@ucd.ie. Orcid: https://orcid.org/0000-0002-0950-3865

For peer review only

ABSTRACT

Introduction: The use of remote monitoring technology to manage the care of COVID-19 patients has been implemented to help reduce the burden placed on healthcare systems during the pandemic and protect the wellbeing of both staff and patients. Remote monitoring allows patients to record their signs and symptoms remotely (e.g., while self-isolating at home) rather than requiring hospitalisation. Healthcare staff can therefore continually monitor their symptoms and be notified when the patient is showing signs of clinical deterioration. However, given the recency of the COVID-19 outbreak, there is a lack of research regarding the effectiveness of remote monitoring interventions to manage COVID-19. This study will aim to evaluate the use of remote monitoring for managing COVID-19 cases from the perspective of both the patient and healthcare staff. **Methods and analysis:** Discharged patients from a large urban teaching hospital in Ireland, who have undergone remote monitoring for COVID-19 will be recruited to take part in a cross-sectional study consisting of a quantitative survey and a qualitative interview. A mixed methods design will be used to understand the experiences of remote monitoring from the perspective of the patient. Healthcare staff who have been involved in the provision of remote monitoring of COVID-19 patients will be recruited to take part in a qualitative interview to understand their experiences with the process. **Ethics and dissemination:** Ethical approval has been granted by the ethical review boards at University College Dublin (UCD) and the and National Research Ethics Committee for COVID-19-related Research (NREC COVID-19). Findings will be disseminated via publications in scientific journals, policy briefs, short reports, and social media.

For peer review only

STRENGTHS AND LIMITATIONS OF THIS STUDY

- A mixed methods approach will allow for an effective evaluation of the remote monitoring programme for the management of COVID-19 patients.
- The perspective of both patients and healthcare staff will be examined, allowing for a more thorough evaluation of remote monitoring programmes for the management of COVID-19.
- The use of structural equation modelling techniques allows us to estimate more accurate parameter estimates in examining the user acceptability of remote monitoring programmes.
- As this study will use a sample of discharged COVID-19 patients and healthcare staff from a hospital in Ireland, these findings may not be generalisable to patients and healthcare staff in other nations, or to patients who present with conditions other than COVID-19.
- This project will use a cross-sectional design; therefore, it will not be possible to infer the temporal ordering among the observed relationships.

For peer review only

INTRODUCTION

Severe acute respiratory syndrome coronavirus 2 (SARS-CoV-2), which causes coronavirus disease 2019 (COVID-19), is a novel virus that, in the majority of clinical manifestations, resembles an influenza-like virus, including similar symptomatology such as cough, headache, fever, and taste and smell disturbances.[1] However, COVID-19 patients can exhibit heterogenous symptom presentations with studies reporting varying symptom profiles in terms of symptom type (e.g., respiratory, neurologic, gastrointestinal) and symptom severity, depending on factors such as age, sex, hospitalisation, comorbid health conditions, and additional risk factors.[2–8] Moreover, COVID-19 appears to follow a biphasic pattern of illness consisting of an initial early viral response phase, followed by an exacerbated inflammatory second phase.[1,9] This second phase can be accompanied by respiratory compromise and hypoxia, including in cases whereby the initial phase was relatively mild in terms of symptom presentation.[10] As such, it is important that COVID-19 patients are continually monitored to ensure that appropriate interventions can take place in the event of clinical deterioration.

However, since the COVID-19 outbreak, healthcare systems have faced numerous challenges, including the loss of resource, mental and physical strain on patients and staff, and staffing shortages due to high levels of COVID-19 among healthcare workers.[11–13] Moreover, individuals may delay seeking treatment [14] for other health conditions due to the fear and anxiety of contracting COVID-19, exacerbating health issues and increasing the subsequent burden on the healthcare system.[15] To effectively protect the wellbeing of patients and healthcare staff, and to prevent the onward transmission of COVID-19, it is imperative that alternative initiatives are implemented to mitigate the impact of the COVID-19 pandemic on healthcare systems. These alternative initiatives may, therefore, allow the limited healthcare resources to be used most efficiently.

For peer review only

One such initiative is the remote monitoring of COVID-19 patients (e.g.,[16–19]). This is a system that involves the use of devices which allow healthcare staff at one location to monitor a patient at a different location. This allows for the home monitoring of patients, including physiological metrics such as oxygen saturation, body temperature, and heart rate,[20] with mild to moderate symptoms (i.e., who do not require hospitalisation) who are in self-isolation, tested positive for COVID-19, and/or are symptomatic. In addition, remote monitoring technology may be useful in monitoring post-acute COVID-19 (or 'long-COVID'), whereby patients continue to exhibit some of the symptoms of COVID-19 despite having recovered.[21,22] Remote monitoring, as an alternative route for providing healthcare, has previously been found to be a useful and cost-effective means to managing patients across numerous types of conditions.[23–27] Recent research has demonstrated the feasibility of using remote monitoring technology to prospectively monitor respiratory illnesses such as common human coronaviruses, rhinovirus, and respiratory syncytial virus.[28] However, given the recency of the COVID-19 pandemic, research pertaining to remote monitoring for managing COVID-19 patients is limited. As such, it is important to examine different aspects of remote monitoring, such as patient and staff experiences and acceptability, to ensure its effectiveness as an alternative means to managing COVID-19 symptoms.

One important aspect to consider is the experience of the technology from the patient's perspective. Patient experience of the intervention is an important aspect in predicting treatment adherence.[29] One such means of modelling patient experience and adherence in remote monitoring-based interventions is through the Technology Acceptance Model (TAM).[30] The TAM posits that the use of technology, such as remote monitoring, is the result of one's behavioural intention to use the technology. Behaviour intention, in turn, can be explained through one's perceived ease-of-use, perceived usefulness, and attitude towards the technology. Alternative models of user acceptance (e.g., the Theory of Planned

For peer review only

Behaviour [TPB] and the Unified Theory of Acceptance and Use of Technology [UTAUT]; see [31,32]) similarly posit that the use of the technology is the result of one's behavioural intention to use the technology. However, in predicting behavioural intention, these models also incorporate factors such as subjective norms/social influences that may play an important role in user acceptance.[31] These models aid in explaining user acceptance by providing a framework to incorporate psychosocial and behaviour theories such as the TPB [33] and the UTAUT,[34] and have been applied to remote monitoring interventions across varying patient groups.[35–38]

Although research on remote monitoring for COVID-19 symptoms is scant, users of remote monitoring technologies for COVID-19 symptoms have generally reported high satisfaction and ease-of-use across interventions.[16,17] Moreover, findings suggest that patient's use of telemedicine systems can be effectively modelled within the TAM and TPB framework.[13,39,40] As such, these frameworks may be extended to modelling adherence in remote monitoring for COVID-19 patients.

Recent evidence, albeit limited, suggests healthcare staff generally have positive experiences with the use of telemedicine during the COVID-19 pandemic and that it may play an important role in their wellbeing.[41,42] For example, healthcare staff reported that they felt the technology was easy to use and were satisfied with the safety precautions implemented to allow them to work from home.[41] Research indicates that healthcare staff generally hold positive views towards the remote monitoring of patients across numerous conditions.[43] However, there are notable concerns across, such as managing increased workload and reduced quality of care from fewer patient visits.[43,44] This suggests that further research is required to better understand the staff experience of remote monitoring technology for COVID-19 patients, particularly across a diverse range of healthcare roles.

For peer review only

This project aims to evaluate a remote monitoring intervention for managing COVID-19 cases in a large urban teaching hospital in Dublin, Ireland. The objectives of this project are twofold: (1) to evaluate the use of remote monitoring from the perspective of the patient, and to identify patient symptom profiles of COVD-19 and post-acute COVID-19 (Work Package 1); and (2) to evaluate the use of remote monitoring from the perspective of the healthcare staff (Work Package 2).

METHODS

Research design

A mixed methods design is proposed to achieve the research objectives of this project. The measures used to collect the data consists of both quantitative measures (reflecting patient experience and medical records) and qualitative interviews (for both patient and staff experience of remote monitoring). Work Package 1 will use both cross-sectional survey data and qualitive interviews to examine patient experiences. Work Package 2 will use qualitative interviews to examine healthcare staff experiences.

Participants and recruitment strategy

Participants will consist of patients and healthcare staff recruited from the clinic site. First, patients who have completed their treatment via remote monitoring will be recruited to gather information on their experiences of the intervention in the form of a quantitative survey. Participant recruitment will begin in February 2021. Participants will be recruited via an email sent through gatekeepers at the clinic site. Those who agree to take part will receive a link to an online survey. A subsample of these participants will also be recruited for a qualitative interview regarding their experiences of remote monitoring. As part of the survey, patients have the option of indicating if they wish to participate in a telephone interview and provide their contact details. These participants will then be contacted by a member of the

For peer review only

research team to arrange a suitable time for interview, once seven days have passed since signing the consent form.

Second, staff who have been remotely monitoring COVID-19 patients will be recruited via an email sent through gatekeepers at the clinic site. These participants will begin to be recruited in February 2021. Those who agree to take part will receive an invitation to participate in a qualitative interview regarding their experiences of remote monitoring from a healthcare staff perspective. If they choose to take part, a member of the research team will contact them to schedule a suitable time for a telephone interview, once seven days have passed since signing the consent form.

The inclusion criteria for this project are that the patient or staff member must (a) be at least 18 years of age, (b) have the capacity to consent, (c) provide their full informed consent to take part in the study, and (d), if they are a member of staff, they must have been monitoring the data and interacting with COVID-19 patients for a minimum of one week.

Measures: Work package 1

Demographic information

Several demographics will be collected including the patient's age (categorised as '18–29', '30–39', '40–49', 50–59', '60+'), sex, education (five categories ranging from 'primary education' to 'degree or postgraduate third level education'), and ethnicity.

COVID-19 symptomatology

Patient experience of COVID-19 will be measured via a 13-item measure that assess the presence, or absence, of COVID-19 symptoms. This measure consists of 12 commonly reported COVID-19 symptoms (fever, cough, shortness of breath or difficulty with breathing, headaches, aches & pains, fatigue/tiredness, nausea, diarrhoea, sore throat, loss of appetite, loss of taste, and loss of smell) and an additional open-ended option for "other". For each

For peer review only

symptom, participants will be asked to indicate whether they experienced the symptom in the first two weeks following their diagnosis.

Post-acute COVID-19 (long-COVID)

To assess for the presence, or absence, of post-acute COVID-19 symptoms, patients will be presented with the same 13-item measure (fever, cough, shortness of breath or difficulty with breathing, headaches, aches & pains, fatigue/tiredness, nausea, diarrhoea, sore throat, loss of appetite, loss of taste, loss of smell and an additional open-ended option for “other”).as the COVID-19 symptomatology questionnaire. However, they will be asked to indicate whether they continued experiencing the symptom for more than two weeks following their diagnosis, and/or are still currently experiencing the symptom.

Pre-existing comorbidities

Pre-exiting comorbidities (i.e., prior to a diagnosis of COVID-19) will be assessed via a nine-item list of physical comorbidities such as chronic respiratory disease and chronic heart disease. Responses will be scored using a trichotomous response format (‘yes’, ‘no’, ‘unknown’).

Prior experience with mobile phone applications

To measure prior experience with smartphone applications, participants will be presented with five statements to which they chose the most appropriate statement (ranging from “I have never used a mobile phone app prior to the COVID-19 app” to “I use mobile phone apps regularly to upload and track my activity”).

Patient experience of remote monitoring equipment

Patients who report using either a pulse oximeter and a remote monitoring phone application, or just the remote monitoring phone application will be asked to complete a 16-item questionnaire pertaining to their experience of the technology. This multidimensional measure is comprised of items relating to the different factors of user technology acceptance,

For peer review only

such as subjective norms and perceived ease-of-use, based on the factors identified from recent meta-analytic research.[31] All items will be rated using a five-point Likert scale (‘strongly disagree’ = 1, ‘strongly agree’ = 5), with higher scores reflecting greater user acceptance towards the remote monitoring technology. Participants will also have the opportunity to share any additional information about their experience through an open-ended question.

Adherence to the use of remote monitoring equipment

Adherence to the use of the pulse oximeter throughout the intervention will be assessed via three statements with participants being asked to choose the statement that best reflects their experience (“I did not use the oximeter”, “I tried to use the oximeter but gave up because it was too difficult”, “I occasionally used the oximeter during my treatment” and “I used the oximeter consistently during my treatment”). In addition, participants will have the option to share any additional information about their experience of using the device through an open-ended question. Adherence to the use of the remote monitoring phone application will be assed via three statements with participants being asked to choose the statement that best reflects their experience (“I did not use the phone application to record my symptoms”, “I tried to use the phone application but gave up because it was too difficult”, “I occasionally used the phone application to record my symptoms throughout my treatment” and “I used the phone application to record my symptoms consistently throughout my treatment”). In addition, participants will have the option to share any additional information about their experience of using the phone application through an open-ended question.

Quality of care

Patient quality of care will be assessed via a 16-item measure (e.g., “he/she respected me as a person”) which represents the care they received during the course of the remote monitoring intervention. All items will be scored using a five-point Likert scale (‘not at all’ =

For peer review only

1, ‘very much’ = 5). Higher scores are indicative of better quality of care (with total scores ranging from 16–80). Additionally, participants will have the option to provide any further information about their care through an open-ended question.

Patient experience interview

Semi-structured interviews will be conducted with patients who have undergone remote monitoring as part of their treatment for COVID-19 (n ≈ 20). These interviews will be conducted by trained interviewers. A topic guide has been designed to explore the patient’s experience of the remote monitoring intervention including their: COVID-19 symptoms; quality of care during the intervention; experience of the remote mentoring equipment; opinion on remote monitoring compared to treatment-as-usual; and opinions on using remote monitoring for other types of conditions.

Data analysis: Work package 1

The first objective will be attained using a mixed-methods design. First, descriptive statistics will assess the overall patient self-reported quality of care, adherence to the use the remote monitoring equipment, and attitudes, perceived usefulness, and perceived ease-of-use towards the remote monitoring technology and provide the sample characteristics.

Second, structure equation modelling will be used to examine different aspects of user acceptance towards remote monitoring technology, such as the perceived usefulness of the equipment from the perspective of the patient. Structural equation modelling is advantageous as it can parse out measurement error thereby yielding more accurate parameter estimates [45] and can be applied to medical research.[46,47] We will also determine the effect of several exogenous covariates such as demographics, pre-existing comorbidities, prior experience with mobile phone applications, COVID-19 symptoms, and quality of care in predicting user acceptance and behavioural intention towards the use of remote monitoring technology.

For peer review only

Third, latent classes, or symptom profiles, of COVID-19 and post-acute COVID-19 will be examined through latent class analysis (LCA), using robust maximum likelihood estimation (MLR). To determine the optimal number of latent classes, models with one to six classes will be examined. To avoid solutions based on local maxima, 500 random sets of starting values will be used followed by 100 final stage optimizations. Several fit indices will be used to determine the fit of each latent class model, including: the Akaike information criterion (AIC),[48] the Bayesian information criterion (BIC),[49] the sample size-adjusted BIC (ssaBIC),[50] entropy values, and the Lo–Mendell–Rubin adjusted likelihood ratio test (LMR-A).[51] Lower AIC, BIC, and ssaBIC values, and higher entropy values, are indicative of better model fit. A non-significant LMR-A value suggests that the model with one less class should be accepted.

Next, to determine the predictors of each symptom profile, a multinomial logistic regression will be performed by regressing the latent classes (identified during the class enumeration process) onto several covariates, using the R3STEP function in Mplus.[52,53] This three-step procedure involves first identifying the most appropriate latent classes; then obtaining the most likely class memberships based on the posterior probabilities of the LCA, while accounting for the classification uncertainty rate (i.e., measurement error); and finally, the most likely class memberships are analysed with the covariates, thereby accounting for at least some of the misclassification error.[53,54]

Fourth, the interviews will be transcribed verbatim. A thematic analysis will be conducted using NVivo to identify common themes throughout the interviews. An inductive approach will be taken to draw out themes by one researcher and will then be reviewed by the research team to ensure all emerging topics are included. A secondary researcher will independently code a subset of transcripts to assess the internal reliability. Themes will be compared across demographic factors such as age and sex.

For peer review only

Measures: Work package 2

Staff experience interview

Semi-structured interviews will be conducted with approximately10 healthcare staff who were responsible for monitoring the data and interacting with COVID-19 patients for a minimum of at least one week at the clinic. These interviews will be conducted by trained interviewers. A topic guide was developed to explore the staff member's experience of the remote monitoring process including: their role in the clinic; the usefulness of equipment such as pulse oximeters; how well the equipment was received by patients; their experiences of the remote monitoring intervention, compared to treatment-as-usual, such as interactions with patients; the impact of remote monitoring on reducing the burden placed on healthcare staff during the pandemic; the benefits and drawback of using remote monitoring; how remote monitoring can be improved; and their opinions on using remote monitoring for other types of conditions.

Data analysis: Work package 2

To achieve the second objective of this project, the data collected during the qualitative interviews with the healthcare staff members will be analysed. These interviews will be transcribed verbatim, and a thematic analysis will be conducted using NVivo to identify common themes throughout the interviews. An inductive approach will be taken to draw out themes by one researcher and will then be reviewed by the research team to ensure all emerging topics are included. A secondary researcher will independently code a subset of transcripts to assess the internal reliability. Themes will be compared across demographic factors such as age and sex.

Patient and public involvement

Patients and/or the public were not involved in the design of this study protocol.

ETHICS AND DISSEMINATION

For peer review only

Ethical approval has been granted by the ethical review boards at University College Dublin (UCD) and the and National Research Ethics Committee for COVID-19-related Research (NREC COVID-19). Data sharing agreements have been put in place between the UCD research team and the hospital. The data will be de-identified and securely transferred to the research team, in accordance with data protection regulations. No identifiable data will be included in the dataset received by the research team at UCD. Online consent will be obtained from the participants of this project.

Findings will be disseminated via publications in scientific journals, policy briefs, short reports, and social media. A summary of the findings will also be shared with participants who informed the research team that they are interested in the results of the project.

For peer review only

Funding Statement

This work was supported by the Science Foundation Ireland (SFI). Grant number: 20/COV/0221.

Competing Interests

None declared.

Authorship Contribution Statement:

RF, SMS, ADB, DJ, and EMcA contributed to the conceptualisation, planning, and design of the project; EMcA obtained funding and ethical approval for the project; RF, SMS, ADB, DJ, and EMcA drafted the initial protocol; EM, TMcA KOR, and EOC provided critical input into the protocol; RF, ADB, SMS, DJ, EM, TMcA KOR, EOC, and EMcA contributed to the writing, revising, and editing of the protocol; All authors contributed to and have approved the final protocol.

For peer review only

REFERENCES

1 Cevik M, Kuppalli K, Kindrachuk J, *et al.* Virology, transmission, and pathogenesis of SARS-CoV-2. *BMJ* 2020;**371**:m3862. doi:10.1136/bmj.m3862

2 Burke RM, Killerby ME, Newton S, *et al.* Symptom profiles of a convenience sample of patients with COVID-19 - United States, January-April 2020. *MMWR Morb Mortal Wkly Rep* 2020;**69**:904–8. doi:10.15585/mmwr.mm6928a2

3 Díaz LA, García-Salum T, Fuentes-López E, *et al.* Symptom profiles and risk factors for hospitalization in patients with SARS-CoV-2 and COVID-19: A large cohort from South America. *Gastroenterology* 2020;**159**:1148–50. doi:10.1053/j.gastro.2020.05.014

4 Gao F, Zheng KI, Wang X-B, *et al.* Obesity is a risk factor for greater COVID-19 severity. *Diabetes Care* 2020;**43**:e72–4. doi:10.2337/dc20-0682

5 Pullen MF, Skipper CP, Hullsiek KH, *et al.* Symptoms of COVID-19 outpatients in the United States. *Open Forum Infect Dis* 2020;**7**. doi:10.1093/ofid/ofaa271

6 Targher G, Mantovani A, Wang X-B, *et al.* Patients with diabetes are at higher risk for severe illness from COVID-19. *Diabetes Metab* 2020;**46**:335–7. doi:10.1016/j.diabet.2020.05.001

7 Yousaf AR, Duca LM, Chu V, *et al.* A prospective cohort study in nonhospitalized household contacts with severe acute respiratory syndrome coronavirus 2 infection: Symptom profiles and symptom change over time. *Clin Infect Dis* Published Online First: 28 July 2020. doi:10.1093/cid/ciaa1072

8 Zhang S-Y, Lian J-S, Hu J-H, *et al.* Clinical characteristics of different subtypes and risk factors for the severity of illness in patients with COVID-19 in Zhejiang, China. *Infect Dis Poverty* 2020;**9**:85. doi:10.1186/s40249-020-00710-6

9 García LF. Immune response, inflammation, and the clinical spectrum of COVID-19.

For peer review only

Front Immunol 2020;**11**. doi:10.3389/fimmu.2020.01441

10 Lescure FX, Bouadma L, Nguyen D, *et al.* Clinical and virological data of the first cases of COVID-19 in Europe: a case series. *Lancet Infect Dis* 2020;**20**:697–706. doi:10.1016/S1473-3099(20)30200-0

11 Adams JG, Walls RM. Supporting the Health Care Workforce During the COVID-19 Global Epidemic. *JAMA* 2020;**323**:1439–40. doi:10.1001/jama.2020.3972

12 Kennelly B, O'Callaghan M, Coughlan D, *et al.* The COVID-19 pandemic in Ireland: An overview of the health service and economic policy response. *Heal Policy Technol* Published Online First: 2020. doi:10.1016/j.hlpt.2020.08.021

13 Ramírez-Correa P, Ramírez-Rivas C, Alfaro-Pérez J, *et al.* Telemedicine acceptance during the COVID-19 pandemic: An empirical example of robust consistent partial least squares path modeling. *Symmetry (Basel)* 2020;**12**. doi:10.3390/sym12101593

14 McDonnell T, Nicholson E, Conlon C, *et al.* Assessing the Impact of COVID-19 Public Health Stages on Paediatric Emergency Attendance. Int. J. Environ. Res. Public Heal. . 2020;**17**. doi:10.3390/ijerph17186719

15 Karacin C, Bilgetekin I, B Basal F, *et al.* How does COVID-19 fear and anxiety affect chemotherapy adherence in patients with cancer. *Futur Oncol* 2020;**16**:2283–93. doi:10.2217/fon-2020-0592

16 Annis T, Pleasants S, Hultman G, *et al.* Rapid implementation of a COVID-19 remote patient monitoring program. *J Am Med Informatics Assoc* 2020;**27**:1326–30. doi:10.1093/jamia/ocaa097

17 Grutters LA, Majoor KI, Mattern ESK, *et al.* Home telemonitoring makes early hospital discharge of COVID-19 patients possible. *J Am Med Informatics Assoc* Published Online First: 15 July 2020. doi:10.1093/jamia/ocaa168

18 Morgan AU, Balachandran M, Do D, *et al.* Remote Monitoring of Patients with

For peer review only

COVID-19: Design, implementation, and outcomes of the first 3,000 patients in COVID Watch. *Nejm Catal Innov Care Deliv* 2020;:10.1056/CAT.20.0342. doi:10.1056/CAT.20.0342

19 O'Carroll O, MacCann R, O'Reilly A, *et al.* Remote monitoring of oxygen saturation in individuals with COVID-19 pneumonia. *Eur Respir J* 2020;**56**:2001492. doi:10.1183/13993003.01492-2020

20 Seshadri DR, Davies E V, Harlow ER, *et al.* Wearable sensors for COVID-19: A call to action to harness our digital infrastructure for remote patient monitoring and virtual assessments. *Front Digit Heal* 2020;**2**:8.https://www.frontiersin.org/article/10.3389/fdgth.2020.00008

21 Cellai M, O'Keefe JB. Characterization of prolonged COVID-19 symptoms in an outpatient telemedicine clinic. *Open Forum Infect Dis* 2020;**7**. doi:10.1093/ofid/ofaa420

22 Mahase E. Covid-19: What do we know about "long covid"? *BMJ* 2020;**370**:m2815. doi:10.1136/bmj.m2815

23 Behar J, Liu C, Kotzen K, *et al.* Remote health diagnosis and monitoring in the time of COVID-19. *Physiol Meas* Published Online First: September 2020. doi:10.1088/1361-6579/abba0a

24 Lopez-Villegas A, Bautista-Mesa R, Peiro S, *et al.* Cost-utility analysis of remote monitoring of users with pacemakers five years after implantation. *Eur J Public Health* 2020;**30**. doi:10.1093/eurpub/ckaa166.568

25 Raatikainen MJP, Uusimaa P, van Ginneken MME, *et al.* Remote monitoring of implantable cardioverter defibrillator patients: a safe, time-saving, and cost-effective means for follow-up. *EP Eur* 2008;**10**:1145–51. doi:10.1093/europace/eun203

26 Schmier JK, Ong KL, Fonarow GC. Cost-effectiveness of remote cardiac monitoring

For peer review only

with the CardioMEMS Heart Failure System. *Clin Cardiol* 2017;**40**:430–6. doi:10.1002/clc.22696

27 Story A, Aldridge RW, Smith CM, *et al.* Smartphone-enabled video-observed versus directly observed treatment for tuberculosis: a multicentre, analyst-blinded, randomised, controlled superiority trial. *Lancet* 2019;**393**:1216–24. doi:10.1016/S0140-6736(18)32993-3

28 Emanuels A, Heimonen J, O'Hanlon J, *et al.* Remote household observation for non-influenza respiratory viral illness. *Clin Infect Dis* Published Online First: November 2020. doi:10.1093/cid/ciaa1719

29 Doyle C, Lennox L, Bell D. A systematic review of evidence on the links between patient experience and clinical safety and effectiveness. *BMJ Open* 2013;**3**:e001570. doi:10.1136/bmjopen-2012-001570

30 Davis FD. Perceived usefulness, perceived ease of use, and user acceptance of information technology. *MIS Q* 1989;**13**:319–40. doi:10.2307/249008

31 Tao D, Wang T, Wang T, *et al.* A systematic review and meta-analysis of user acceptance of consumer-oriented health information technologies. *Comput Human Behav* 2020;**104**:106147. doi:10.1016/j.chb.2019.09.023

32 Weaver MS, Lukowski J, Wichman B, *et al.* Human connection and technology connectivity: A systematic review of available telehealth survey instruments. *J Pain Symptom Manage* Published Online First: 2020. doi:10.1016/j.jpainsymman.2020.10.010

33 Ajzen I. *Attitudes, personality, and behavior.* Homewood, IL, US: : Dorsey Press 1988.

34 Venkatesh V, Morris MG, Davis GB, *et al.* User acceptance of information technology: Toward a unified view. *MIS Q* 2003;**27**:425–78. doi:10.2307/30036540

For peer review only

35 Giger JT, Pope ND, Vogt HB, *et al.* Remote patient monitoring acceptance trends among older adults residing in a frontier state. *Comput Human Behav* 2015;**44**:174–82. doi:10.1016/j.chb.2014.11.044

36 Kohnke A, Cole ML, Bush R. Incorporating UTAUT predictors for understanding home care patients' and clinician's acceptance of healthcare telemedicine equipment. *J Technol Manag Innov* 2014;**9**:29–41. doi:10.4067/S0718-27242014000200003

37 Sin DYE, Guo X, Yong DWW, *et al.* Assessment of willingness to Tele-monitoring interventions in patients with type 2 diabetes and/or hypertension in the public primary healthcare setting. *BMC Med Inform Decis Mak* 2020;**20**:11. doi:10.1186/s12911-020-1024-4

38 Tsai CH. The adoption of a telehealth system: The integration of extended technology acceptance model and health belief model. *J Med Imaging Heal Informatics* 2014;**4**:448–55. doi:10.1166/jmihi.2014.1278

39 Jen WY, Hung MC. An empirical study of adopting mobile healthcare service: The family's perspective on the healthcare needs of their elderly members. *Telemed e-Health* 2010;**16**:41–8. doi:10.1089/tmj.2009.0093

40 Zhang X, Han X, Dang Y, *et al.* User acceptance of mobile health services from users' perspectives: The role of self-efficacy and response-efficacy in technology acceptance. *Informatics Heal Soc Care* 2017;**42**:194–206. doi:10.1080/17538157.2016.1200053

41 Lu A, Cannesson M, Kamdar N. The tipping point of medical technology: Implications for the postpandemic era. *Anesth Analg* 2020;**131**:335–9. doi:10.1213/ANE.0000000000005040

42 Moazzami B, Razavi-Khorasani N, Dooghaie Moghadam A, *et al.* COVID-19 and telemedicine: Immediate action required for maintaining healthcare providers well-being. *J Clin Virol* 2020;**126**:104345. doi:10.1016/j.jcv.2020.104345

For peer review only

43 Davis MM, Freeman M, Kaye J, *et al.* A systematic review of clinician and staff views on the acceptability of incorporating remote monitoring technology into primary care. *Telemed J E-Health* 2014;**20**:428–38. doi:10.1089/tmj.2013.0166

44 Jacob C, Sanchez-Vazquez A, Ivory C. Social, Organizational, and Technological Factors Impacting Clinicians' Adoption of Mobile Health Tools: Systematic Literature Review. *JMIR Mhealth Uhealth* 2020;**8**:e15935. doi:10.2196/15935

45 Bollen KA. *Structural equations with latent variables*. New York: : Wiley 1989.

46 Bentler PM, Stein JA. Structural equation models in medical research. *Stat Methods Med Res* 1992;**1**:159–81. doi:10.1177/096228029200100203

47 Waters KA, Mast BT, Vella S, *et al.* Structural equation modeling of sleep apnea, inflammation, and metabolic dysfunction in children. *J Sleep Res* 2007;**16**:388–95. doi:10.1111/j.1365-2869.2007.00614.x

48 Akaike H. Factor analysis and AIC. *Psychometrika* 1987;**52**:317–32. doi:10.1007/BF02294359

49 Schwarz G. Estimating the Dimension of a Model. *Ann Stat* 1978;**6**:461–4. doi:10.1214/aos/1176344136

50 Sclove SL. Application of model-selection criteria to some problems in multivariate analysis. *Psychometrika* 1987;**52**:333–43.

51 Lo Y, Mendell NR, Rubin DB. Testing the number of components in a normal mixture. *Biometrika* 2001;**88**:767–78. doi:10.1093/biomet/88.3.767

52 Muthén LK, Muthén BO. *Mplus User's Guide*. 8th ed. Los Angeles, CA: : Muthén & Muthén 2018.

53 Vermunt JK. Latent class modeling with covariates: Two improved three-step approaches. *Polit Anal* 2010;**18**:450–69. doi:10.1093/pan/mpq025

54 Asparouhov T, Muthén B. Auxiliary variables in mixture modeling: Three-step

For peer review only

approaches using Mplus. *Struct Equ Model A Multidiscip J* 2014;**21**:329–41. doi:10.1080/10705511.2014.915181

For peer review only

STROBE Statement—Checklist of items that should be included in reports of ***cross-sectional studies***

	Item No	Recommendation	Page No
**Title and abstract**	1	(*a*) Indicate the study's design with a commonly used term in the title or the abstract	2
		(*b*) Provide in the abstract an informative and balanced summary of what was done and what was found	2
**Introduction**			
Background/rationale	2	Explain the scientific background and rationale for the investigation being reported	4-6
Objectives	3	State specific objectives, including any prespecified hypotheses	7
**Methods**			
Study design	4	Present key elements of study design early in the paper	7
Setting	5	Describe the setting, locations, and relevant dates, including periods of recruitment, exposure, follow-up, and data collection	7-8
Participants	6	(*a*) Give the eligibility criteria, and the sources and methods of selection of participants	7-8
Variables	7	Clearly define all outcomes, exposures, predictors, potential confounders, and effect modifiers. Give diagnostic criteria, if applicable	8-11
Data sources/ measurement	8*	For each variable of interest, give sources of data and details of methods of assessment (measurement). Describe comparability of assessment methods if there is more than one group	8-11
Bias	9	Describe any efforts to address potential sources of bias	11-12
Study size	10	Explain how the study size was arrived at	7-8
Quantitative variables	11	Explain how quantitative variables were handled in the analyses. If applicable, describe which groupings were chosen and why	11-12
Statistical methods	12	(*a*) Describe all statistical methods, including those used to control for confounding	11-12
		(*b*) Describe any methods used to examine subgroups and interactions	11-13
		(*c*) Explain how missing data were addressed	11-12
		(*d*) If applicable, describe analytical methods taking account of sampling strategy	11-12
		(*e*) Describe any sensitivity analyses	
**Results**			
Participants	13*	(a) Report numbers of individuals at each stage of study—eg numbers potentially eligible, examined for eligibility, confirmed eligible, included in the study, completing follow-up, and analysed	NA
		(b) Give reasons for non-participation at each stage	NA
		(c) Consider use of a flow diagram	
Descriptive data	14*	(a) Give characteristics of study participants (eg demographic, clinical, social) and information on exposures and potential confounders	NA
		(b) Indicate number of participants with missing data for each variable of interest	MA
Outcome data	15*	Report numbers of outcome events or summary measures	NA

For peer review only

Main results	16	(*a*) Give unadjusted estimates and, if applicable, confounder-adjusted estimates and their precision (eg, 95% confidence interval). Make clear which confounders were adjusted for and why they were included	NA
		(*b*) Report category boundaries when continuous variables were categorized	NA
		(*c*) If relevant, consider translating estimates of relative risk into absolute risk for a meaningful time period	NA
Other analyses	17	Report other analyses done—eg analyses of subgroups and interactions, and sensitivity analyses	NA
**Discussion**			
Key results	18	Summarise key results with reference to study objectives	NA
Limitations	19	Discuss limitations of the study, taking into account sources of potential bias or imprecision. Discuss both direction and magnitude of any potential bias	3
Interpretation	20	Give a cautious overall interpretation of results considering objectives, limitations, multiplicity of analyses, results from similar studies, and other relevant evidence	NA
Generalisability	21	Discuss the generalisability (external validity) of the study results	NA
**Other information**			
Funding	22	Give the source of funding and the role of the funders for the present study and, if applicable, for the original study on which the present article is based	15

*Give information separately for exposed and unexposed groups.

Note: An Explanation and Elaboration article discusses each checklist item and gives methodological background and published examples of transparent reporting. The STROBE checklist is best used in conjunction with this article (freely available on the Web sites of PLoS Medicine at http://www.plosmedicine.org/, Annals of Internal Medicine at http://www.annals.org/, and Epidemiology at http://www.epidem.com/). Information on the STROBE Initiative is available at www.strobe-statement.org.

For peer review only

BMJ Open

A mixed methods protocol to examine the acceptability and clinical characteristics of a remote monitoring programme for delivery of COVID-19 care, among healthcare staff and patients.

Journal:	*BMJ Open*
Manuscript ID	bmjopen-2021-051408.R1
Article Type:	Protocol
Date Submitted by the Author:	14-Jun-2021
Complete List of Authors:	Fox, Robert; University College Dublin, School of Nursing, Midwifery & Health Systems<br>Symmons, Sophie Mulcahy; University College Dublin, School of Nursing, Midwifery & Health Systems<br>De Brún, Aoife; University College Dublin, School of Nursing, Midwifery & Health Systems<br>Joyce, David; University College Dublin, School of Nursing, Midwifery & Health Systems<br>Muldoon, Eavan G; Mater Misericordiae University Hospital, Dept of Infectious Diseases; University College Dublin, School of Medicine<br>McGinty, Tara; Mater Misericordiae University Hospital, Infectious Diseases; University College Dublin, School of Medicine<br>O'Reilly, Katherine M.A.; University College Dublin, School of Medicine; Mater Misericordiae University Hospital, Department of Respiratory Medicine<br>O’Connor, Eileen; Mater Misericordiae University Hospital, Dept of Infectious Diseases<br>McAuliffe, Eilish; University College Dublin, School of Nursing, Midwifery and Health Systems
**Primary Subject Heading**:	Public health
Secondary Subject Heading:	Health informatics, Health services research
Keywords:	COVID-19, Telemedicine < BIOTECHNOLOGY & BIOINFORMATICS, PUBLIC HEALTH

SCHOLARONE™
Manuscripts

I, the Submitting Author has the right to grant and does grant on behalf of all authors of the Work (as defined in the below author licence), an exclusive licence and/or a non-exclusive licence for contributions from authors who are: i) UK Crown employees; ii) where BMJ has agreed a CC-BY licence shall apply, and/or iii) in accordance with the terms applicable for US Federal Government officers or employees acting as part of their official duties; on a worldwide, perpetual, irrevocable, royalty-free basis to BMJ Publishing Group Ltd ("BMJ") its licensees and where the relevant Journal is co-owned by BMJ to the co-owners of the Journal, to publish the Work in this journal and any other BMJ products and to exploit all rights, as set out in our licence.

The Submitting Author accepts and understands that any supply made under these terms is made by BMJ to the Submitting Author unless you are acting as an employee on behalf of your employer or a postgraduate student of an affiliated institution which is paying any applicable article publishing charge ("APC") for Open Access articles. Where the Submitting Author wishes to make the Work available on an Open Access basis (and intends to pay the relevant APC), the terms of reuse of such Open Access shall be governed by a Creative Commons licence – details of these licences and which Creative Commons licence will apply to this Work are set out in our licence referred to above.

Other than as permitted in any relevant BMJ Author's Self Archiving Policies, I confirm this Work has not been accepted for publication elsewhere, is not being considered for publication elsewhere and does not duplicate material already published. I confirm all authors consent to publication of this Work and authorise the granting of this licence.

For peer review only

A mixed methods protocol to examine the acceptability and clinical characteristics of a remote monitoring programme for delivery of COVID-19 care, among healthcare staff and patients.

Robert Fox[a], Sophie Mulcahy Symmons[a], Aoife DeBrun[a], David Joyce[a], Eavan G Muldoon[b,c], Tara McGinty[b,c], Katherine M.A.O'Reilly [c,d] Eileen O'Connor[b], Eilish McAuliffe[a]

[a]Centre for Interdisciplinary Research, Education and Innovation in Health Systems (IRIS), School of Nursing, Midwifery & Health Systems, University College Dublin

[b]Dept of Infectious Diseases, Mater Misericordiae University Hospital, Dublin

[c]School of Medicine, University College Dublin

d Department of Respiratory Medicine, Mater Misericordiae University Hospital

Author Note

Corresponding author: Robert Fox, Centre for Interdisciplinary Research, Education and Innovation in Health Systems (IRIS), School of Nursing, Midwifery & Health Systems, University College Dublin, Dublin, Ireland.
Email: Robert.Fox@ucd.ie. Orcid: https://orcid.org/0000-0002-0950-3865

For peer review only

ABSTRACT

Introduction: The use of remote monitoring technology to manage the care of COVID-19 patients has been implemented to help reduce the burden placed on healthcare systems during the pandemic and protect the wellbeing of both staff and patients. Remote monitoring allows patients to record their signs and symptoms remotely (e.g., while self-isolating at home) rather than requiring hospitalisation. Healthcare staff can therefore continually monitor their symptoms and be notified when the patient is showing signs of clinical deterioration. However, given the recency of the COVID-19 outbreak, there is a lack of research regarding the acceptance of remote monitoring interventions to manage COVID-19. This study will aim to evaluate the use of remote monitoring for managing COVID-19 cases from the perspective of both the patient and healthcare staff. **Methods and analysis:** Discharged patients from a large urban teaching hospital in Ireland, who have undergone remote monitoring for COVID-19 will be recruited to take part in a cross-sectional study consisting of a quantitative survey and a qualitative interview. A mixed methods design will be used to understand the experiences of remote monitoring from the perspective of the patient. Healthcare staff who have been involved in the provision of remote monitoring of COVID-19 patients will be recruited to take part in a qualitative interview to understand their experiences with the process. Structural equation modelling will be used to examine the acceptance of the remote monitoring technology. Latent class analysis will be used to identify COVID-19 symptom profiles. Interview data will be examined using thematic analysis. **Ethics and dissemination:** Ethical approval has been granted by the ethical review boards at University College Dublin (UCD) and the and National Research Ethics Committee for COVID-19-related Research (NREC COVID-19). Findings will be disseminated via publications in scientific journals, policy briefs, short reports, and social media.

For peer review only

STRENGTHS AND LIMITATIONS OF THIS STUDY

- A mixed methods approach will allow for an in-depth examination of the remote monitoring programme for the management of COVID-19 patients.
- The perspective of both patients and healthcare staff will be examined, allowing for a more thorough evaluation of remote monitoring programmes for the management of COVID-19.
- The use of structural equation modelling techniques allows us to estimate more accurate parameter estimates in examining the user acceptability of remote monitoring programmes.
- As this study will use a sample of discharged COVID-19 patients and healthcare staff from a hospital in Ireland, these findings may not be generalisable to patients and healthcare staff in other nations, or to patients who present with conditions other than COVID-19.
- This project will use a cross-sectional design; therefore, it will not be possible to infer the temporal ordering among the observed relationships.

For peer review only

INTRODUCTION

Severe acute respiratory syndrome coronavirus 2 (SARS-CoV-2), which causes coronavirus disease 2019 (COVID-19), is a virus that, in the majority of clinical manifestations, resembles an influenza-like virus, including similar symptomatology such as cough, headache, fever, and taste and smell disturbances.[1] However, COVID-19 patients can exhibit heterogenous symptom presentations with studies reporting varying symptom profiles in terms of symptom type (e.g., respiratory, neurologic, gastrointestinal) and symptom severity, depending on factors such as age, sex, hospitalisation, comorbid health conditions, and additional risk factors.[2–8] Moreover, COVID-19 appears to follow a biphasic pattern of illness consisting of an initial early viral response phase, followed by an exacerbated inflammatory second phase.[1,9] This second phase can be accompanied by respiratory compromise and hypoxia, including in cases whereby the initial phase was relatively mild in terms of symptom presentation.[10] As such, it is important that COVID-19 patients are continually monitored to ensure that appropriate interventions can take place in the event of clinical deterioration.

However, since the COVID-19 outbreak, healthcare systems have faced numerous challenges, including the loss of resource, mental and physical strain on patients and staff, and staffing shortages due to high levels of COVID-19 among healthcare workers.[11–13] Moreover, individuals may delay seeking treatment [14] for other health conditions due to the fear and anxiety of contracting COVID-19, exacerbating health issues and increasing the subsequent burden on the healthcare system.[15] To effectively protect the wellbeing of patients and healthcare staff, and to prevent the onward transmission of COVID-19, it is imperative that alternative initiatives are implemented to mitigate the impact of the COVID-19 pandemic on healthcare systems. These alternative initiatives may, therefore, allow the limited healthcare resources to be used most efficiently.

For peer review only

One such initiative is the remote monitoring of COVID-19 patients (e.g.,[16–19]). This is a system that involves the use of devices which allow healthcare staff at one location to monitor a patient at a different location. This allows for the home monitoring of patients, including physiological metrics such as oxygen saturation, body temperature, and heart rate,[20] with mild to moderate symptoms (i.e., who do not require hospitalisation) who are in self-isolation, tested positive for COVID-19, and/or are symptomatic. In addition, remote monitoring technology may be useful in monitoring post-acute COVID-19 (or 'long-COVID'), whereby patients continue to exhibit some of the symptoms of COVID-19 despite having recovered.[21,22] Remote monitoring, as an alternative route for providing healthcare, has previously been found to be a useful and cost-effective means to managing patients across numerous types of conditions.[23–27] Recent research has demonstrated the feasibility of using remote monitoring technology to prospectively monitor respiratory illnesses such as common human coronaviruses, rhinovirus, and respiratory syncytial virus.[28] However, given the recency of the COVID-19 pandemic, research pertaining to remote monitoring for managing COVID-19 patients is limited. As such, it is important to examine different aspects of remote monitoring, such as patient and staff experiences and acceptability, to ensure its effectiveness as an alternative means to managing COVID-19 symptoms.

One important aspect to consider is the experience of the technology from the patient's perspective. Patient experience of the intervention is an important aspect in predicting treatment adherence.[29] One such means of modelling patient experience and adherence in remote monitoring-based interventions is through the Technology Acceptance Model (TAM).[30] The TAM posits that the use of technology, such as remote monitoring, is the result of one's behavioural intention to use the technology. Behaviour intention, in turn, can be explained through one's perceived ease-of-use, perceived usefulness, and attitude towards the technology. Alternative models of user acceptance (e.g., the Theory of Planned

For peer review only

Behaviour [TPB], the Unified Theory of Acceptance and Use of Technology [UTAUT], and the Task-Technology Fit [TTF] models; see [31–33]) similarly posit that the use of the technology is the result of one's behavioural intention to use the technology. However, in predicting behavioural intention, these models also incorporate factors such as subjective norms/social influences that may play an important role in user acceptance.[31] These models aid in explaining user acceptance by providing a framework to incorporate psychosocial and behaviour theories such as the TPB [34] and the UTAUT,[35] and have been applied to remote monitoring interventions across varying patient groups.[36–39]

Although research on remote monitoring for COVID-19 symptoms is scant, users of remote monitoring technologies for COVID-19 symptoms have generally reported high satisfaction and ease-of-use across interventions.[16,17] Moreover, findings suggest that patient's use of telemedicine systems can be effectively modelled within the TAM and TPB framework.[13,40,41] As such, these frameworks may be extended to modelling adherence in remote monitoring for COVID-19 patients.

Recent evidence, albeit limited, suggests healthcare staff generally have positive experiences with the use of telemedicine during the COVID-19 pandemic and that it may play an important role in their wellbeing.[42,43] For example, healthcare staff reported that they felt the technology was easy to use and were satisfied with the safety precautions implemented to allow them to work from home.[42] Research indicates that healthcare staff generally hold positive views towards the remote monitoring of patients across numerous conditions.[44] However, there are notable concerns across, such as managing increased workload and reduced quality of care from fewer patient visits.[44,45] This suggests that further research is required to better understand the staff experience of remote monitoring technology for COVID-19 patients, particularly across a diverse range of healthcare roles.

For peer review only

This project aims to evaluate a remote monitoring intervention for managing COVID-19 cases in a large urban teaching hospital in Dublin, Ireland. The objectives of this project are twofold. First, the acceptance of the remote monitoring technology from the perspective of the patient (objective 1a) and healthcare staff (objective 1b) will be examined using a mixed methods approach. User acceptance from the perspective of the patient will be examined using factors extracted from an integrated model of the UTAUT and TTF models (see [33]), while also controlling for a number of covariates, using quantitative analyses. User acceptance of remote monitoring for managing COVID-19 patients from the perspective of both the patient and healthcare staff will also be examined using a qualitative methodology.

Second, patient symptom profiles of COVID-19 and post-acute COVID-19 will be identified using a quantitative methods approach (objective 2). Identifying patient symptom profiles of COVID-19 and post-acute COVID-19 may highlight potential subgroups of patients who may be more likely to present with higher symptomatology or different sets of co-occurring symptoms. In addition, predictors, such as demographic variables and pre-existing comorbidities, of the COVID-19 symptom profiles will be determined. Identifying these predictors may be of particular benefit to identifying patients who may need to be monitored for a longer duration. For example, identifying patients who are likely to remain symptomatic (particularly symptoms such as shortness of breath) may need to be monitored for a longer duration after acute COVID-19. Furthermore, identifying such profiles may aid COVID-19 positive patients in being prepared for a likely set of symptoms that they may experience long-term, depending on the predictors of the COVID-19 symptom profiles. For example, identifying patients who are likely to have persistent symptoms such as fatigue and loss of taste/smell may be useful in helping the patient being prepared for these long-term effects.

METHODS

For peer review only

Research design

A mixed methods design is proposed to achieve the research objectives of this project. The measures used to collect the data consists of both quantitative measures (e.g., user acceptance of the remote monitoring technology) and qualitative interviews (for both patient and staff experience of remote monitoring). Work Package 1 will use both quantitative cross-sectional survey data and qualitive interviews to examine patient experiences. Work Package 1 will address objectives 1a and 2. Quantitative analyses will be used to determine the acceptance of the remote monitoring technology among the patients, and to identify the patient symptom profiles of COVID-19 and post-acute COVID-19. Work Package 2 will use qualitative interviews to examine healthcare staff experiences. Work package 2 will address objective 1b.

Participants and recruitment strategy

Participants will consist of patients and healthcare staff recruited from the clinic site. First, patients who have completed their treatment via remote monitoring will be recruited to gather information on their experiences of the intervention in the form of a quantitative survey. Patient recruitment will begin in February 2021 and end in June 2021. A total of 925 patients have been discharged from the remote monitoring service. Patients will be recruited via an email sent through gatekeepers at the clinic site. All patients will receive an email with the Participant Information sheet and a link to an online survey. A subsample of these patients will also be recruited for a qualitative interview regarding their experiences of remote monitoring. As part of the survey, patients have the option of indicating if they wish to participate in a telephone interview and provide their contact details. Patients who consented to interview will be purposively sampled to get a representative sample based on age, gender, and education. These patients will then be contacted by a member of the research

For peer review only

team to arrange a suitable time for interview, once seven days have passed since signing the consent form.

Second, staff who have been remotely monitoring COVID-19 patients will be recruited via an email sent through gatekeepers at the clinic site. These participants will begin to be recruited in February 2021 and end in June 2021. A total of 25 staff worked in the virtual clinic since it was set up in April 2020. All staff who worked in the clinic will receive an invitation to participate in a qualitative interview regarding their experiences of remote monitoring from a healthcare staff perspective. If they choose to take part, a member of the research team will contact them to schedule a suitable time for a telephone interview, once seven days have passed since signing the consent form.

The inclusion criteria for this project are that the patient or staff member must (a) be at least 18 years of age, (b) have the capacity to consent, (c) provide their full informed consent to take part in the study, (d) if they are a patient, they must have received a positive COVID-19 diagnosis, (e) if they are a patient, they must have undergone treatment via remote monitoring, and (f), if they are a member of staff, they must have been monitoring the data and interacting with COVID-19 patients for a minimum of one week. Patients who did not receive a positive diagnosis for COVID-19 and/or did not undergo treatment via remote monitoring will be excluded. The exclusion criterion for staff members is if they worked at the virtual clinic for less than one week.

Measures: Work package 1

Demographic information

Several demographics will be collected including the patient's age (categorised as '18–29', '30–39', '40–49', 50–59', '60+'), sex, education (five categories ranging from 'primary education' to 'degree or postgraduate third level education'), and ethnicity.

COVID-19 symptomatology

For peer review only

Patient experience of COVID-19 will be measured via a 13-item measure that assess the presence, or absence, of COVID-19 symptoms. This measure consists of 12 commonly reported COVID-19 symptoms (fever, cough, shortness of breath or difficulty with breathing, headaches, aches & pains, fatigue/tiredness, nausea, diarrhoea, sore throat, loss of appetite, loss of taste, and loss of smell) and an additional open-ended option for “other”. For each symptom, participants will be asked to indicate whether they experienced the symptom in the first two weeks following their diagnosis.

Post-acute COVID-19 (long-COVID)

To assess for the presence, or absence, of post-acute COVID-19 symptoms, patients will be presented with the same 13-item measure (fever, cough, shortness of breath or difficulty with breathing, headaches, aches & pains, fatigue/tiredness, nausea, diarrhoea, sore throat, loss of appetite, loss of taste, loss of smell and an additional open-ended option for “other”).as the COVID-19 symptomatology questionnaire. However, they will be asked to indicate whether they continued experiencing the symptom for more than two weeks following their diagnosis, and/or are still currently experiencing the symptom.

Pre-existing comorbidities

Pre-exiting comorbidities (i.e., prior to a diagnosis of COVID-19) will be assessed via a nine-item list of physical comorbidities such as chronic respiratory disease and chronic heart disease. Responses will be scored using a trichotomous response format (‘yes’, ‘no’, ‘unknown’).

Prior experience with mobile phone applications

To measure prior experience with smartphone applications, participants will be presented with five statements to which they chose the most appropriate statement (ranging from “I have never used a mobile phone app prior to the COVID-19 app” to “I use mobile phone apps regularly to upload and track my activity”).

For peer review only

User acceptance of remote monitoring equipment

Patients who report using either a pulse oximeter and a remote monitoring phone application, or just the remote monitoring phone application will be asked to complete a 16-item questionnaire pertaining to their experience of the technology. This multidimensional measure is comprised of items relating to the different factors of user technology acceptance, based on a recent integrated model consisting of factors from the UTAUT and TTF models (see [33]). Several factors will be extracted from this model, consisting of 'social influence' (two items; the degree to which important others agree to the use of the technology; e.g., "people who influence my behaviour thought it was important that I use the remote monitoring equipment"), 'facilitating conditions', (three items; individual's perception of the availability of resources to use the technology; e.g., "I felt I had the necessary knowledge to use the remote monitoring equipment"), 'effort expectancy' (four items; ease-of-use related to using the technology; e.g., "I find it easy to use such equipment"), 'performance expectancy'(four items; effectiveness to users in performing specific tasks; e.g., "I feel remote monitoring equipment is useful in obtaining health information"), and 'task-technology fit' (three items; the degree to which users believe that the performance of the technology match its intended use; e.g., "In general, the remote monitoring equipment fully met my needs"). All items will be rated using a five-point Likert scale ('strongly disagree' = 1, 'strongly agree' = 5), with higher scores reflecting greater scores towards its respective factor. Previous psychometric research has supported the validity and reliability of these items.[33] Participants will also have the opportunity to share any additional information about their experience through an open-ended question.

Adherence to the use of remote monitoring equipment

Adherence to the use of the pulse oximeter throughout the intervention will be assessed via three statements with participants being asked to choose the statement that best

For peer review only

reflects their experience ("I did not use the oximeter", "I tried to use the oximeter but gave up because it was too difficult", "I occasionally used the oximeter during my treatment" and "I used the oximeter consistently during my treatment"). In addition, participants will have the option to share any additional information about their experience of using the device through an open-ended question. Adherence to the use of the remote monitoring phone application will be assed via three statements with participants being asked to choose the statement that best reflects their experience ("I did not use the phone application to record my symptoms", "I tried to use the phone application but gave up because it was too difficult", "I occasionally used the phone application to record my symptoms throughout my treatment" and "I used the phone application to record my symptoms consistently throughout my treatment"). In addition, participants will have the option to share any additional information about their experience of using the phone application through an open-ended question.

Perceived patient-centred care (patient-professional interaction)

Perceived patient-centred care will be assessed via the 16-item Patient-Professional Interaction Questionnaire (PPIQ).[46] The PPIQ examines patient-centred care by healthcare professional from the perspective of the patient by evaluating different aspects of the integration between the healthcare professional and the patient. This measure consists of four factors (each comprised of four items), which represents the care they received during the course of the remote monitoring intervention: effective communication (e.g., "he/she provided me with clear information"), interest in the patient's agenda (e.g., "he/she was interested in what I want from care"), empathy (e.g., "he/she understood my emotions"), and patient involvement in care (e.g., "he/she gave me time to ask and to talk about the illness". All items will be scored using a five-point Likert scale ('not at all' = 1, 'very much' = 5). Higher scores are indicative of better patient-centred care. The psychometric attributes of this

For peer review only - http://bmjopen.bmj.com/site/about/guidelines.xhtml

measure have previously been supported.[46] Additionally, participants will have the option to provide any further information about their care through an open-ended question.

Patient experience interview

Semi-structured interviews will be conducted with patients who have undergone remote monitoring as part of their treatment for COVID-19 (n ≈ 20, or until saturation is reached). These interviews will be conducted by trained interviewers. A topic guide has been designed to explore the patient's experience of the remote monitoring intervention including their: experience of having COVID-19; interactions with staff during the intervention; experience of the remote mentoring equipment; opinion on remote monitoring compared to treatment-as-usual; and opinions on using remote monitoring for other types of conditions. The interviews will take approximately 20 minutes. Interviews will be conducted via telephone on loudspeaker and recorded using audio-recorder or via video call on Zoom and the video call recorded to facilitate COVID-19 restrictions.

Data analysis: Work package 1

The first objective will be attained using a mixed-methods design. First, descriptive statistics will assess the overall patient self-reported patient-professional interaction, adherence to the use the remote monitoring equipment, and acceptance towards the remote monitoring technology and provide the sample characteristics.

Second, structure equation modelling will be used to examine different aspects of user acceptance towards remote monitoring technology, such as the performance expectancy of the equipment from the perspective of the patient. Structural equation modelling is advantageous as it can parse out measurement error thereby yielding more accurate parameter estimates [47] and can be applied to medical research.[48,49] We will also determine the effect of several exogenous covariates such as demographics, pre-existing comorbidities, prior experience with mobile phone applications, and patient-professional interaction in

For peer review only

predicting user acceptance towards the use of remote monitoring technology. Given the larger sample size required for structure equation modelling, in the event that an insufficient sample size is gathered, then a multiple regression analyse will be conducted instead.

Third, latent classes, or symptom profiles, of COVID-19 and post-acute COVID-19 will be examined through latent class analysis (LCA), using robust maximum likelihood estimation (MLR). To determine the optimal number of latent classes, models with one to six classes will be examined. To avoid solutions based on local maxima, 500 random sets of starting values will be used followed by 100 final stage optimizations. Several fit indices will be used to determine the fit of each latent class model, including: the Akaike information criterion (AIC),[50] the Bayesian information criterion (BIC),[51] the sample size-adjusted BIC (ssaBIC),[52] entropy values, and the Lo–Mendell–Rubin adjusted likelihood ratio test (LMR-A).[53] Lower AIC, BIC, and ssaBIC values, and higher entropy values, are indicative of better model fit. A non-significant LMR-A value suggests that the model with one less class should be accepted. In the event that an insufficient sample size is gathered, then a cluster analysis will be conducted instead.

Next, to determine the predictors of each symptom profile, a multinomial logistic regression will be performed by regressing the latent classes (identified during the class enumeration process) onto several covariates, using the R3STEP function in Mplus.[54,55] This three-step procedure involves first identifying the most appropriate latent classes; then obtaining the most likely class memberships based on the posterior probabilities of the LCA, while accounting for the classification uncertainty rate (i.e., measurement error); and finally, the most likely class memberships are analysed with the covariates, thereby accounting for at least some of the misclassification error.[55,56]

Fourth, the interviews will be transcribed verbatim. A thematic analysis will be conducted using NVivo version 12 [57] to identify common themes throughout the

For peer review only

interviews. An inductive approach will be taken to draw out themes, without a pre-existing theory in the literature or based on the researchers' preconceptions, by one researcher through familiarisation with the data, initial coding, refinement and subsequently grouped into themes to best represent the data. It will then be reviewed by the research team to ensure all emerging topics are included and consensus is reached. A secondary researcher will independently code a subset of transcripts to assess the internal reliability.

Measures: Work package 2

Staff experience interview

Semi-structured interviews will be conducted with up to 15 (or until saturation in the data is reached) healthcare staff who were responsible for monitoring the data and interacting with COVID-19 patients for a minimum of at least one week at the clinic and agree to take part in the study. These interviews will be conducted by trained interviewers and will take approximately 30 minutes. Interviews will be conducted over the phone and recorded using audio-recorder or via video call on Zoom and the video call recorded. A topic guide was developed to explore the staff member's experience of the remote monitoring process including: their role in the clinic; the usefulness of equipment such as pulse oximeters; how well the equipment was received by patients; their experiences of the remote monitoring intervention compared to treatment-as-usual, such as interactions with patients; the impact of remote monitoring on reducing the burden placed on healthcare staff during the pandemic; the benefits and drawback of using remote monitoring; how remote monitoring can be improved; and their opinions on using remote monitoring for other types of conditions.

Data analysis: Work package 2

To achieve the second objective of this project, the data collected during the qualitative interviews with the healthcare staff members will be analysed. These interviews will be transcribed verbatim, and a thematic analysis will be conducted using NVivo to

For peer review only

identify common themes throughout the interviews. An inductive approach will be taken to draw out themes by one researcher and will then be reviewed by the research team to ensure all emerging topics are included, following the same steps as Work package 1. A secondary researcher will independently code a subset of transcripts to assess the internal reliability.

Patient and public involvement

Patients and/or the public were not involved in the design of this study protocol.

ETHICS AND DISSEMINATION

Ethical approval has been granted by the National Research Ethics Committee for COVID-19-related Research (NREC COVID-19; reference number: 20-NREC-COV-093). Data sharing agreements have been put in place between the UCD research team and the hospital. The data will be de-identified and securely transferred to the research team, in accordance with data protection regulations. No identifiable data will be included in the dataset received by the research team at UCD. Online consent will be obtained from the participants of this project.

Findings will be disseminated via publications in scientific journals, policy briefs, short reports, and social media. A summary of the findings will also be shared with participants who informed the research team that they are interested in the results of the project.

For peer review only

Funding Statement

This work was supported by the Science Foundation Ireland (SFI). Grant number: 20/COV/0221.

Competing Interests

None declared.

Authorship Contribution Statement:

RF, SMS, ADB, DJ, and EMcA contributed to the conceptualisation, planning, and design of the project; EMcA obtained funding and ethical approval for the project; RF, SMS, ADB, DJ, and EMcA drafted the initial protocol; EM, TMcA KOR, and EOC provided critical input into the protocol; RF, ADB, SMS, DJ, EM, TMcA KOR, EOC, and EMcA contributed to the writing, revising, and editing of the protocol; All authors contributed to and have approved the final protocol.

For peer review only

REFERENCES

1 Cevik M, Kuppalli K, Kindrachuk J, *et al.* Virology, transmission, and pathogenesis of SARS-CoV-2. *BMJ* 2020;371:m3862. doi:10.1136/bmj.m3862

2 Burke RM, Killerby ME, Newton S, *et al.* Symptom profiles of a convenience sample of patients with COVID-19 - United States, January-April 2020. *MMWR Morb Mortal Wkly Rep* 2020;69:904–8. doi:10.15585/mmwr.mm6928a2

3 Díaz LA, García-Salum T, Fuentes-López E, *et al.* Symptom profiles and risk factors for hospitalization in patients with SARS-CoV-2 and COVID-19: A large cohort from South America. *Gastroenterology* 2020;159:1148–50. doi:10.1053/j.gastro.2020.05.014

4 Gao F, Zheng KI, Wang X-B, *et al.* Obesity is a risk factor for greater COVID-19 severity. *Diabetes Care* 2020;43:e72–4. doi:10.2337/dc20-0682

5 Pullen MF, Skipper CP, Hullsiek KH, *et al.* Symptoms of COVID-19 outpatients in the United States. *Open Forum Infect Dis* 2020;7. doi:10.1093/ofid/ofaa271

6 Targher G, Mantovani A, Wang X-B, *et al.* Patients with diabetes are at higher risk for severe illness from COVID-19. *Diabetes Metab* 2020;46:335–7. doi:10.1016/j.diabet.2020.05.001

7 Yousaf AR, Duca LM, Chu V, *et al.* A prospective cohort study in nonhospitalized household contacts with severe acute respiratory syndrome coronavirus 2 infection: Symptom profiles and symptom change over time. *Clin Infect Dis* 2020. doi:10.1093/cid/ciaa1072

8 Zhang S-Y, Lian J-S, Hu J-H, *et al.* Clinical characteristics of different subtypes and risk factors for the severity of illness in patients with COVID-19 in Zhejiang, China. *Infect Dis Poverty* 2020;9:85. doi:10.1186/s40249-020-00710-6

9 García LF. Immune response, inflammation, and the clinical spectrum of COVID-19.

For peer review only

Front Immunol 2020;11. doi:10.3389/fimmu.2020.01441

10 Lescure FX, Bouadma L, Nguyen D, *et al.* Clinical and virological data of the first cases of COVID-19 in Europe: a case series. *Lancet Infect Dis* 2020;20:697–706. doi:10.1016/S1473-3099(20)30200-0

11 Adams JG, Walls RM. Supporting the Health Care Workforce During the COVID-19 Global Epidemic. *JAMA* 2020;323:1439–40. doi:10.1001/jama.2020.3972

12 Kennelly B, O'Callaghan M, Coughlan D, *et al.* The COVID-19 pandemic in Ireland: An overview of the health service and economic policy response. *Heal Policy Technol* 2020. doi:10.1016/j.hlpt.2020.08.021

13 Ramírez-Correa P, Ramírez-Rivas C, Alfaro-Pérez J, *et al.* Telemedicine acceptance during the COVID-19 pandemic: An empirical example of robust consistent partial least squares path modeling. *Symmetry (Basel)* 2020;12. doi:10.3390/sym12101593

14 McDonnell T, Nicholson E, Conlon C, *et al.* Assessing the Impact of COVID-19 Public Health Stages on Paediatric Emergency Attendance. Int. J. Environ. Res. Public Heal. . 2020;17. doi:10.3390/ijerph17186719

15 Karacin C, Bilgetekin I, B Basal F, *et al.* How does COVID-19 fear and anxiety affect chemotherapy adherence in patients with cancer. *Futur Oncol* 2020;16:2283–93. doi:10.2217/fon-2020-0592

16 Annis T, Pleasants S, Hultman G, *et al.* Rapid implementation of a COVID-19 remote patient monitoring program. *J Am Med Informatics Assoc* 2020;27:1326–30. doi:10.1093/jamia/ocaa097

17 Grutters LA, Majoor KI, Mattern ESK, *et al.* Home telemonitoring makes early hospital discharge of COVID-19 patients possible. *J Am Med Informatics Assoc* 2020. doi:10.1093/jamia/ocaa168

18 Morgan AU, Balachandran M, Do D, *et al.* Remote Monitoring of Patients with

For peer review only

COVID-19: Design, implementation, and outcomes of the first 3,000 patients in COVID Watch. *Nejm Catal Innov Care Deliv* 2020;:10.1056/CAT.20.0342. doi:10.1056/CAT.20.0342

19 O'Carroll O, MacCann R, O'Reilly A, *et al.* Remote monitoring of oxygen saturation in individuals with COVID-19 pneumonia. *Eur Respir J* 2020;56:2001492. doi:10.1183/13993003.01492-2020

20 Seshadri DR, Davies E V, Harlow ER, *et al.* Wearable sensors for COVID-19: A call to action to harness our digital infrastructure for remote patient monitoring and virtual assessments. *Front Digit Heal* 2020;2:8.https://www.frontiersin.org/article/10.3389/fdgth.2020.00008

21 Cellai M, O'Keefe JB. Characterization of prolonged COVID-19 symptoms in an outpatient telemedicine clinic. *Open Forum Infect Dis* 2020;7. doi:10.1093/ofid/ofaa420

22 Mahase E. Covid-19: What do we know about "long covid"? *BMJ* 2020;370:m2815. doi:10.1136/bmj.m2815

23 Behar J, Liu C, Kotzen K, *et al.* Remote health diagnosis and monitoring in the time of COVID-19. *Physiol Meas* 2020. doi:10.1088/1361-6579/abba0a

24 Lopez-Villegas A, Bautista-Mesa R, Peiro S, *et al.* Cost-utility analysis of remote monitoring of users with pacemakers five years after implantation. *Eur J Public Health* 2020;30. doi:10.1093/eurpub/ckaa166.568

25 Raatikainen MJP, Uusimaa P, van Ginneken MME, *et al.* Remote monitoring of implantable cardioverter defibrillator patients: a safe, time-saving, and cost-effective means for follow-up. *EP Eur* 2008;10:1145–51. doi:10.1093/europace/eun203

26 Schmier JK, Ong KL, Fonarow GC. Cost-effectiveness of remote cardiac monitoring with the CardioMEMS Heart Failure System. *Clin Cardiol* 2017;40:430–6.

For peer review only

doi:10.1002/clc.22696

27 Story A, Aldridge RW, Smith CM, *et al.* Smartphone-enabled video-observed versus directly observed treatment for tuberculosis: a multicentre, analyst-blinded, randomised, controlled superiority trial. *Lancet* 2019;393:1216–24. doi:10.1016/S0140-6736(18)32993-3

28 Emanuels A, Heimonen J, O'Hanlon J, *et al.* Remote household observation for non-influenza respiratory viral illness. *Clin Infect Dis* 2020. doi:10.1093/cid/ciaa1719

29 Doyle C, Lennox L, Bell D. A systematic review of evidence on the links between patient experience and clinical safety and effectiveness. *BMJ Open* 2013;3:e001570. doi:10.1136/bmjopen-2012-001570

30 Davis FD. Perceived usefulness, perceived ease of use, and user acceptance of information technology. *MIS Q* 1989;13:319–40. doi:10.2307/249008

31 Tao D, Wang T, Wang T, *et al.* A systematic review and meta-analysis of user acceptance of consumer-oriented health information technologies. *Comput Human Behav* 2020;104:106147. doi:10.1016/j.chb.2019.09.023

32 Weaver MS, Lukowski J, Wichman B, *et al.* Human connection and technology connectivity: A systematic review of available telehealth survey instruments. *J Pain Symptom Manage* 2020. doi:10.1016/j.jpainsymman.2020.10.010

33 Wang H, Tao D, Yu N, *et al.* Understanding consumer acceptance of healthcare wearable devices: An integrated model of UTAUT and TTF. *Int J Med Inform* 2020;139:104156. doi:10.1016/j.ijmedinf.2020.104156

34 Ajzen I. *Attitudes, personality, and behavior.* Homewood, IL, US: Dorsey Press 1988.

35 Venkatesh V, Morris MG, Davis GB, *et al.* User acceptance of information technology: Toward a unified view. *MIS Q* 2003;27:425–78. doi:10.2307/30036540

For peer review only

36 Giger JT, Pope ND, Vogt HB, *et al.* Remote patient monitoring acceptance trends among older adults residing in a frontier state. *Comput Human Behav* 2015;44:174–82. doi:10.1016/j.chb.2014.11.044

37 Kohnke A, Cole ML, Bush R. Incorporating UTAUT predictors for understanding home care patients' and clinician's acceptance of healthcare telemedicine equipment. *J Technol Manag Innov* 2014;9:29–41. doi:10.4067/S0718-27242014000200003

38 Sin DYE, Guo X, Yong DWW, *et al.* Assessment of willingness to Tele-monitoring interventions in patients with type 2 diabetes and/or hypertension in the public primary healthcare setting. *BMC Med Inform Decis Mak* 2020;20:11. doi:10.1186/s12911-020-1024-4

39 Tsai CH. The adoption of a telehealth system: The integration of extended technology acceptance model and health belief model. *J Med Imaging Heal Informatics* 2014;4:448–55. doi:10.1166/jmihi.2014.1278

40 Jen WY, Hung MC. An empirical study of adopting mobile healthcare service: The family's perspective on the healthcare needs of their elderly members. *Telemed e-Health* 2010;16:41–8. doi:10.1089/tmj.2009.0093

41 Zhang X, Han X, Dang Y, *et al.* User acceptance of mobile health services from users' perspectives: The role of self-efficacy and response-efficacy in technology acceptance. *Informatics Heal Soc Care* 2017;42:194–206. doi:10.1080/17538157.2016.1200053

42 Lu A, Cannesson M, Kamdar N. The tipping point of medical technology: Implications for the postpandemic era. *Anesth Analg* 2020;131:335–9. doi:10.1213/ANE.0000000000005040

43 Moazzami B, Razavi-Khorasani N, Dooghaie Moghadam A, *et al.* COVID-19 and telemedicine: Immediate action required for maintaining healthcare providers well-being. *J Clin Virol* 2020;126:104345. doi:10.1016/j.jcv.2020.104345

For peer review only

44 Davis MM, Freeman M, Kaye J, *et al.* A systematic review of clinician and staff views on the acceptability of incorporating remote monitoring technology into primary care. *Telemed J E-Health* 2014;20:428–38. doi:10.1089/tmj.2013.0166

45 Jacob C, Sanchez-Vazquez A, Ivory C. Social, Organizational, and Technological Factors Impacting Clinicians' Adoption of Mobile Health Tools: Systematic Literature Review. *JMIR Mhealth Uhealth* 2020;8:e15935. doi:10.2196/15935

46 Casu G, Gremigni P, Sommaruga M. The Patient-Professional Interaction Questionnaire (PPIQ) to assess patient centered care from the patient's perspective. *Patient Educ Couns* 2019;102:126–33. doi:10.1016/j.pec.2018.08.006

47 Bollen KA. *Structural equations with latent variables*. New York: Wiley 1989.

48 Bentler PM, Stein JA. Structural equation models in medical research. *Stat Methods Med Res* 1992;1:159–81. doi:10.1177/096228029200100203

49 Waters KA, Mast BT, Vella S, *et al.* Structural equation modeling of sleep apnea, inflammation, and metabolic dysfunction in children. *J Sleep Res* 2007;16:388–95. doi:10.1111/j.1365-2869.2007.00614.x

50 Akaike H. Factor analysis and AIC. *Psychometrika* 1987;52:317–32. doi:10.1007/BF02294359

51 Schwarz G. Estimating the Dimension of a Model. *Ann Stat* 1978;6:461–4. doi:10.1214/aos/1176344136

52 Sclove SL. Application of model-selection criteria to some problems in multivariate analysis. *Psychometrika* 1987;52:333–43.

53 Lo Y, Mendell NR, Rubin DB. Testing the number of components in a normal mixture. *Biometrika* 2001;88:767–78. doi:10.1093/biomet/88.3.767

54 Muthén LK, Muthén BO. *Mplus User's Guide*. 8th ed. Los Angeles, CA: Muthén & Muthén 2018.

For peer review only

55 Vermunt JK. Latent class modeling with covariates: Two improved three-step approaches. *Polit Anal* 2010;18:450–69. doi:10.1093/pan/mpq025

56 Asparouhov T, Muthén B. Auxiliary variables in mixture modeling: Three-step approaches using Mplus. *Struct Equ Model A Multidiscip J* 2014;21:329–41. doi:10.1080/10705511.2014.915181

57 QSR International Pty Ltd. NVivo (version 12). 2020.https://www.qsrinternational.com/nvivo-qualitative-data-analysis-software/home

For peer review only

STROBE Statement—Checklist of items that should be included in reports of ***cross-sectional studies***

	Item No	Recommendation	Page No
**Title and abstract**	1	(*a*) Indicate the study's design with a commonly used term in the title or the abstract	2
		(*b*) Provide in the abstract an informative and balanced summary of what was done and what was found	2
**Introduction**			
Background/rationale	2	Explain the scientific background and rationale for the investigation being reported	4-7
Objectives	3	State specific objectives, including any prespecified hypotheses	7
**Methods**			
Study design	4	Present key elements of study design early in the paper	8
Setting	5	Describe the setting, locations, and relevant dates, including periods of recruitment, exposure, follow-up, and data collection	8
Participants	6	(*a*) Give the eligibility criteria, and the sources and methods of selection of participants	9
Variables	7	Clearly define all outcomes, exposures, predictors, potential confounders, and effect modifiers. Give diagnostic criteria, if applicable	9-13
Data sources/ measurement	8*	For each variable of interest, give sources of data and details of methods of assessment (measurement). Describe comparability of assessment methods if there is more than one group	9-13
Bias	9	Describe any efforts to address potential sources of bias	13-15
Study size	10	Explain how the study size was arrived at	8-9
Quantitative variables	11	Explain how quantitative variables were handled in the analyses. If applicable, describe which groupings were chosen and why	13-14
Statistical methods	12	(*a*) Describe all statistical methods, including those used to control for confounding	13-14
		(*b*) Describe any methods used to examine subgroups and interactions	13-15
		(*c*) Explain how missing data were addressed	13-14
		(*d*) If applicable, describe analytical methods taking account of sampling strategy	13-14
		(*e*) Describe any sensitivity analyses	
**Results**			
Participants	13*	(a) Report numbers of individuals at each stage of study—eg numbers potentially eligible, examined for eligibility, confirmed eligible, included in the study, completing follow-up, and analysed	NA
		(b) Give reasons for non-participation at each stage	NA
		(c) Consider use of a flow diagram	
Descriptive data	14*	(a) Give characteristics of study participants (eg demographic, clinical, social) and information on exposures and potential confounders	NA
		(b) Indicate number of participants with missing data for each variable of interest	MA
Outcome data	15*	Report numbers of outcome events or summary measures	NA

For peer review only - http://bmjopen.bmj.com/site/about/guidelines.xhtml

Main results	16	(*a*) Give unadjusted estimates and, if applicable, confounder-adjusted estimates and their precision (eg, 95% confidence interval). Make clear which confounders were adjusted for and why they were included	NA
		(*b*) Report category boundaries when continuous variables were categorized	NA
		(*c*) If relevant, consider translating estimates of relative risk into absolute risk for a meaningful time period	NA
Other analyses	17	Report other analyses done—eg analyses of subgroups and interactions, and sensitivity analyses	NA
**Discussion**			
Key results	18	Summarise key results with reference to study objectives	NA
Limitations	19	Discuss limitations of the study, taking into account sources of potential bias or imprecision. Discuss both direction and magnitude of any potential bias	3
Interpretation	20	Give a cautious overall interpretation of results considering objectives, limitations, multiplicity of analyses, results from similar studies, and other relevant evidence	NA
Generalisability	21	Discuss the generalisability (external validity) of the study results	NA
**Other information**			
Funding	22	Give the source of funding and the role of the funders for the present study and, if applicable, for the original study on which the present article is based	17

*Give information separately for exposed and unexposed groups.

Note: An Explanation and Elaboration article discusses each checklist item and gives methodological background and published examples of transparent reporting. The STROBE checklist is best used in conjunction with this article (freely available on the Web sites of PLoS Medicine at http://www.plosmedicine.org/, Annals of Internal Medicine at http://www.annals.org/, and Epidemiology at http://www.epidem.com/). Information on the STROBE Initiative is available at www.strobe-statement.org.

For peer review only

For peer review only - http://bmjopen.bmj.com/site/about/guidelines.xhtml

	Item	Guide questions/description	Response
**Domain 1: Research team and reflexivity**			
Personal Characteristics			
1.	Interviewer/facilitator	Which author/s conducted the interview or focus group?	Sophie Mulcahy Symmons
2.	Credentials	What were the researcher's credentials? *E.g. PhD, MD*	MSc
3.	Occupation	What was their occupation at the time of the study?	Research assistant
4.	Gender	Was the researcher male or female?	Female
5.	Experience and training	What experience or training did the researcher have?	Conducted qualitive interviews on research projects with patients, healthcare staff and researchers in the health sciences

Relationship with participants			
6.	Relationship established	Was a relationship established prior to study commencement?	No prior relationship exists between the interviewer and participant
7.	Participant knowledge of the interviewer	What did the participants know about the researcher? e.g. *personal goals, reasons for doing the research*	Participants will be aware of the aims and purpose of the study via the Participant Information Leaflet and brief before the interview. However, they will not be aware of any personal details relating to the interviewer.
8.	Interviewer characteristics	What characteristics were reported about the interviewer/facilitator? e.g. *Bias, assumptions, reasons and interests in the research topic*	No characteristics will be reported relating to the interviewer.
**Domain 2: study design**			
Theoretical framework			

9.	Methodological orientation and Theory	What methodological orientation was stated to underpin the study? *e.g. grounded theory, discourse analysis, ethnography, phenomenology, content analysis*	An inductive approach will be taken to draw out themes, without a pre-existing theory. A thematic analysis will be used to analyse the data.
Participant selection			
10.	Sampling	How were participants selected? *e.g. purposive, convenience, consecutive, snowball*	Purposive sampling will be used based on age, sex, and education for patients. Convenience sampling will be used for staff
11.	Method of approach	How were participants approached? e.*g. face-to-face, telephone, mail, email*	Participants will be approached via email
12.	Sample size	How many participants were in the study?	Up to 15 staff members and 20 patients will be included in the study, or until saturation in the data is reached
13.	Non-participation	How many people refused to participate or dropped out? Reasons?	N/A
Setting			

14.	Setting of data collection	Where was the data collected? e.*g. home, clinic, workplace*	As the data will be collected using either telephone or video calls, the setting was a convenient location for the participant
15.	Presence of non-participants	Was anyone else present besides the participants and researchers?	No one else will be present during the interview
16.	Description of sample	What are the important characteristics of the sample? *e.g. demographic data, date*	Staff: Must have been monitoring the data and interacting with COVID-19 patients for a minimum of one week in the clinic<br>Patient: Must have received a positive COVID-19 diagnosis and undergone care via remote monitoring
Data collection			
17.	Interview guide	Were questions, prompts, guides provided by the authors? Was it pilot tested?	Yes, guides will be provided by the authors.<br>No pilot test will be conducted.
18.	Repeat interviews	Were repeat interviews carried out? If yes, how many?	No

19.	Audio/visual recording	Did the research use audio or visual recording to collect the data?	Audio recording
20.	Field notes	Were field notes made during and/or after the interview or focus group?	Yes
21.	Duration	What was the duration of the interviews or focus group?	Approximately 30 minutes for staff.<br>Approximately 20 minutes for patients.
22.	Data saturation	Was data saturation discussed?	Yes.
23.	Transcripts returned	Were transcripts returned to participants for comment and/or correction?	No.
**Domain 3: analysis and findings**z			
Data analysis			
24.	Number of data coders	How many data coders coded the data?	Two will code the data

25.	Description of the coding tree	Did authors provide a description of the coding tree?	N/A
26.	Derivation of themes	Were themes identified in advance or derived from the data?	Themes will be derived from the data
27.	Software	What software, if applicable, was used to manage the data?	NVIVo version 12
28.	Participant checking	Did participants provide feedback on the findings?	No.
Reporting			
29.	Quotations presented	Were participant quotations presented to illustrate the themes / findings? Was each quotation identified? e.*g. participant number*	N/A
30.	Data and findings consistent	Was there consistency between the data presented and the findings?	N/A
31.	Clarity of major themes	Were major themes clearly presented in the findings?	N/A

32.	Clarity of minor themes	Is there a description of diverse cases or discussion of minor themes?	N/A

For peer review only
